# Analyses of *MADS-box* Genes Suggest *HvMADS56* to Regulate Lateral Spikelet Development in Barley

**DOI:** 10.3390/plants10122825

**Published:** 2021-12-20

**Authors:** Mohammed A. Sayed, Mohamed Allam, Quinn Kalby Heck, Ieva Urbanavičiūtė, Twan Rutten, David Stuart, Shakhira Zakhrabekova, Andreas Börner, Klaus Pillen, Mats Hansson, Helmy M. Youssef

**Affiliations:** 1 Leibniz Institute of Plant Genetics and Crop Plant Research (IPK), Corrensstr. 3, OT Gatersleben, 06466 Seeland, Germany; msayed@aun.edu.eg (M.A.S.); rutten@ipk-gatersleben.de (T.R.); boerner@ipk-gatersleben.de (A.B.); 2 Faculty of Agriculture, Assuit University, Assuit 71526, Egypt; mohamed.allam@studenti.unitus.it; 3 Department of Agricultural and Forest Sciences, Tuscia University, Via S. C. de Lellis, snc, 01100 Viterbo, Italy; ievaurbanaviciute@yahoo.com; 4 Department of Biology, Lund University, Sölvegatan 35B, 22362 Lund, Sweden; quinn@plen.ku.dk (Q.K.H.); david.stuart@biol.lu.se (D.S.); shakhira.zakhrabekova@biol.lu.se (S.Z.); mats.hansson@biol.lu.se (M.H.); 5 Institute of Agricultural and Nutritional Sciences, Faculty of Natural Sciences III, Martin Luther University Halle-Wittenberg, 06120 Halle, Germany; klaus.pillen@landw.uni-halle.de; 6 Faculty of Agriculture, Cairo University, Giza 12613, Egypt

**Keywords:** *MADS-box* family, spike development, lateral spikelet formation, *Hordeum vulgare*, phylogenetic tree

## Abstract

MADS-box transcription factors are crucial regulators of inflorescence and flower development in plants. Therefore, the recent interest in this family has received much attention in plant breeding programs due to their impact on plant development and inflorescence architecture. The aim of this study was to investigate the role of *HvMADS-box* genes in lateral spikelet development in barley (*Hordeum vulgare* L.). A set of 30 spike-contrasting barley lines were phenotypically and genotypically investigated under controlled conditions. We detected clear variations in the spike and spikelet development during the developmental stages among the tested lines. The lateral florets in the *deficiens* and *semi-deficiens* lines were more reduced than in two-rowed cultivars except *cv.* Kristina. Interestingly, *cv.* Kristina, *int-h.43* and *int-i.39* exhibited the same behavior as *def.5, def.6, semi-def.1, semi-def.8* regarding development and showed reduced lateral florets size. In HOR1555, HOR7191 and HOR7041, the lateral florets continued their development, eventually setting seeds. In contrast, lateral florets in two-rowed barley stopped differentiating after the awn primordia stage giving rise to lateral floret sterility. At harvest, the lines tested showed large variation for all central and lateral spikelet-related traits. Phylogenetic analysis showed that more than half of the 108 *MADS-box* genes identified are highly conserved and are expressed in different barley tissues. Re-sequence analysis of a subset of these genes showed clear polymorphism in either SNPs or in/del. Variation in *HvMADS56* correlated with altered lateral spikelet morphology. This suggests that *HvMADS56* plays an important role in lateral spikelet development in barley.

## 1. Introduction

Increasing the yield performance of cereal crops remains one of the major goals of plant breeding programs [1]. One of the strategies has been to increase the number of seed primordia per inflorescence. In rice [2], maize [3] and to a certain extent also in wheat [4,5], this has been achieved by enhancing the number of florets. In barley (*Hordeum vulgare* L.), however, grain number has been increased by suppressing floret sterility [6]. The inflorescence architecture of barley is unique among the Triticeae. The barley inflorescence is an unbranched spike [7], whose constituent units are called spikelets. Each spikelet contains a single floret and two glumes and is attached to the floral axis (the rachis). Floral patterning of the three spikelet primordia of immature barley inflorescence—one central and two laterals—is initiated at each rachis node. In wild barley (*H. vulgare* sp. *spontaneum*) and domesticated barley of the two-rowed type (*H. vulgare* sp. *vulgare*), lateral floret development is arrested, and only the central spikelets set grains [8].

Altering the fertility of lateral florets is one of the major strategies to increase the number of grains at each rachis node. It is likely that six-rowed barley (*H. vulgare* convar. *hexastichon*), with three fertile spikelets per rachis node, was derived from an ancestral two-rowed form (convar. *distichon* (L.) Alef.) [6]. Based on the central and lateral spikelet fertility, barley plants are categorized into four different groups [9]: (i) two-rowed barley having a fertile central spikelet flanked by two sterile lateral ones, (ii) deficiens barley, basically being a two-rowed barley in which the lateral spikelets are absent or extremely reduced; (iii) six-rowed barley having three fully fertile spikelets per node; (iv) labile-barley having the lateral spikelets fully developed or absent, fertile or sterile, even within one individual spike. Another row-type class named intermedium barley (*H. vulgare* convar. *intermedium* (Körn.) Mansf. (syn. *H. intermedium* Körn.)) displays various degrees of lateral spikelet fertility and seed development intermediate between two- and six-rowed types [6]. Another group of barley row-type, the so-called *semi-deficiens*-barley, has a two-rowed phenotype whose reduced lateral spikelets are longer than those in *deficiens* but smaller than those in true two-rowed barley.

The barley row-type is regulated by several loci of which some are identified; *Six-rowed spike 1* (*Vrs1* [syn = *HvHox1*], [7], *Vrs2* [10], *Vrs3* [11,12], *Vrs4* [13], *intermedium spike-c* (*int-c* [syn = *Vrs5*], [14]. Out of these loci, four encode transcription factors; *Vrs1* (homeodomain-leucine zipper class I (corresponds to HD-Zip I in Arabidopsis)), *Vrs2* (SHORT INTERNODES), *Vrs4* (RAMOSA2) and *Vrs5* (TEOSINTE BRANCHED1), while *Vrs3* encodes an enzyme (a histone H3K9 demethylase). Previous genetic analyses of the intermedium barley have revealed that other genes, independently of *Vrs1*, can increase the size of florets and even stimulate occasional grain setting in lateral spikelets [6]. The results showed that the six-rowed phenotype arises in various panels of intermedium barley carrying the two-rowed allele of *Vrs1* in the presence of the six-rowed allele of *Int-c*, previously considered only as a modifier of lateral spikelet fertility. The six-rowed allele of *Int-c* probably arose before domestication and is associated with the enlargement of lateral florets in wild barley. Since this allele cannot overcome the lateral florets sterility in the genomic background of wild barleys, we infer the existence of other loci at which novel alleles or allelic combinations were selected for after domestication to increase the grain number of barley independently of *Vrs1* [6]. Our understanding of the genetic basis of barley inflorescence development and growth has greatly increased over the recent decades. The spikelet arrangement patterning in two-rowed and wild barleys is regulated by the transcriptional regulator (*Vrs2*), which plays a role in controlling the levels of important developmental hormones along the spike [10,15,16].

Among the various transcription factors active in biological systems is the MADS-box family. The name is derived from MCM1 (in yeast), AG (in Arabidopsis), DEFICIENS (in Antirrhinum), and SRF (in mammals). These four protein families are considered as the first four discovered transcription factors [17,18]. Many of the *MADS-box* genes are crucial for floral initiation, development and growth and proposed to be the dynamic force of the floral diversity in many plants [19,20]. Therefore, a better understanding of *MADS-box* gene function can provide information on how different floral structures evolved and identify target genes for the improvement of crop breeding programs [21]. In Arabidopsis, most of the floral organs development genes encode *MADS-box* transcription factors. The *MADS-box* genes in rice, maize and barley showed crucial similarities to those in Arabidopsis, suggesting a similar function in floral development [22,23].

In the present work, we studied the relations between *HvMADS-box* genes and the phenotypic development of lateral spikelets using a set of spike-variation barley lines. We found that *HvMADS56* could be considered as a novel gene that plays an important role in lateral spikelet development in barley.

## 2. Results

### 2.1. Phenotypic Status and Scanning Electron Microscopy (SEM) of the Tested Barley Lines

Thirty barley lines with a large variation in their spike morphology were selected to investigate the role of *HvMADS-box* genes in relation to their spike and spikelet development. Among these lines were two-rowed, deficiens, semi-deficiens and intermedium types. The 30 barley lines were grown under controlled greenhouse conditions. At harvest, plant height, number of tillers per plant, number of spikes per plant, main culm spike length and weight as well as central and lateral spikelet length, width, weight and area were measured (Table S1). The analysis of variance revealed highly significant differences for all studied traits. The coefficient of determination (R^2^) and heritability in broad-sense (H_b_) ranged from 84.7 to 90.9% (for central seed width) and from 93.9 to 96.8% (for central seed area), respectively (Table S2). Plant height ranged between 59 cm in *def.8* and 128 cm in HOR6407, with an average of 83 cm. Number of tillers per plant varied from 2.3 in *int-l.81* to 22.0 in *cv.* Bonus with an average of 10.6. Number of spikes per plant ranged from 2.0 in *int-l.81* to 21.3 in *cv.* Bonus with an average of 9.7. Spike length ranged from 4.4 cm in HOR10166 to 11.8 cm in *cv.* Bonus with an average of 9.2 cm. Spike weight varied from 0.34 g in *def.5* to 2.84 g in HOR7191 with an average of 1.27 g. The seed weight of the central spikelet ranged between 0.12 g in *def.5* and 1.45 g in *cv.* Bonus with an average of 0.87 g. Seed width of the central spikelet ranged between 3.17 mm in *def.6* and 4.20 mm in *cv.* Bowman with an average of 3.68 mm. Seed length of the central spikelet ranged between 6.77 mm in HOR1555 and 16.30 mm in HOR6407 with an average of 10.45 mm. Seed area of the central spikelet varied between 18.13 mm^2^ in HOR1555 and 37.90 mm^2^ in *int-l.81* with an average of 26.0 mm^2^. Seed numbers of the central spikelet ranged between 4.0 seeds in *int-l.81* and 27.3 seeds in *cv.* Bonus with an average of 16.7 seeds. Thousand seed weight of the central spikelet ranged between 28.8 g in *def.6* and 70.1 g in *int-l.81* with an average of 52.2 g (Table S1).

Four lines showed fertile lateral spikelets and were setting seeds; *hex-v.3*, HOR1555, HOR7191 and HOR7041. All the other lines showed sterile lateral spikelets. Highly significant differences were observed for lateral spikelet weight (LSW), width (LSWi), length (LSL), area (LSA), and thousand seeds weight (in case of fertile lateral spikelet) or thousand spikelet weight (in case of sterile lateral spikelet) (TSW) (Table 1). Large estimates of H_b_ and R^2^ were obtained for all lateral spikelet-related traits (Table S2). Among the sterile lateral spikelets, the genotype *def.2* showed the lowest values of lateral spikelet weight, spikelet width, spikelet length, spikelet area and TSW recording 0.00007 g, 0.54 mm, 3.46 mm, 0.97 mm^2^ and 0.07 g, respectively (Table 1). The genotype *int*-*e.4* gave the highest values of spikelet weight, spikelet width, spikelet area and TSW recording 0.0026 g, 2.28 mm, 13.99 mm^2^ and 2.59 g, respectively. While the genotype *int-f.19* gave the longest spikelet by length of 12.58 mm.

Among the four lines showing fertile lateral spikelets, *hex-v.3*, despite displaying the longest lateral spikelets (11.22 mm), exhibited the lowest values of lateral spikelet weight, width, area and TSW by 0.0224 g, 3.15 mm, 19.04 mm^2^ and 22.37 g, respectively (Table 1). The genotype HOR7191 showed the highest values for spikelet weight, width, area and TSW by 0.0477 g, 4.23 mm, 27.93 mm^2^ and 47.74 g, respectively. HOR1555 genotype gave the shortest spikelet by value of 9.77 mm.

**Figure 1:**
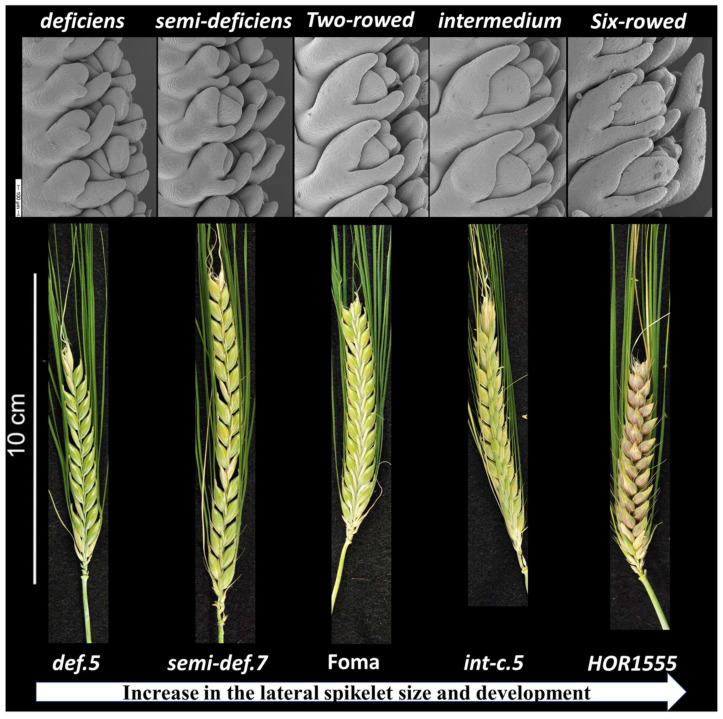
SEM of lateral spikelet development at awn primordia stage and spike form showing increase in the lateral spikelet size and development from left to right in the lines; *deficiens* phenotype (*cv.* Kristina, *int-h.43, int-i39, def.5, def.6, semi-def.1, semi-def.8*), *semi-deficiens* phenotype (*semi-def.4, semi-def.5 and semi-def.7*), two-rowed phenotype (*cvs.* Bonus, Bowman and Foma), *intermedium* phenotype (mutants *int-a.1, int-c.5, int-e.20, int-e.4, int-f.19, int-l.81, int-m.*85, HOR6211, HOR10166 and HOR6407) and Six-rowed phenotype (*hex-v.3,* HOR1555, HOR7191 and HOR7041).

For SEM analysis, for each individual line, five or more spikes were dissected from the developmental stages triple mound, glum primordia, stamen primordia, lemma primordia and awn primordia [24] from each of the lines. We detected clear variation among the tested lines (Figure 1). The lateral florets in the *deficiens* lines were more reduced than the *semi-deficiens* and two-rowed cultivars such as *cv.* Bonus. Interestingly, *cv.* Kristina, *int-h.43 and int-i.39* showed the same appearance as *def.5, def.6, semi-def.1, semi-def.8* in development and reduced lateral floret size. Up until the awn primordium stage, the lateral spikelets of the six-rowed lines HOR1555, HOR7191 and HOR7041 developed similar to those of two-rowed cultivars. After the awn primordia stage, however, lateral spikelets of these HOR lines continued their development, while those of two-rowed barley stopped differentiating at this stage, leading to lateral floret sterility.

### 2.2. HvMADS-box Genes Map

**Figure 2:**
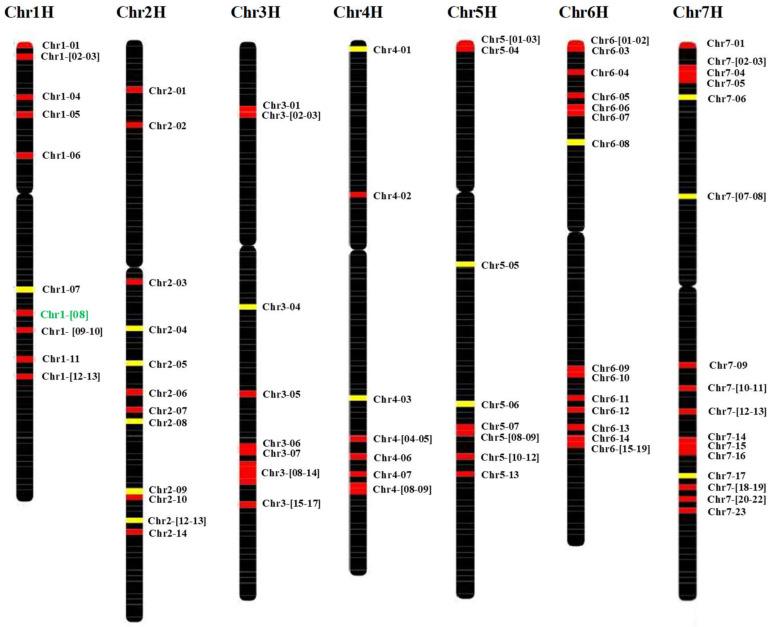
Genetic map of the *HvMADS-box* genes. The map includes all seven barley chromosomes. Genes are indicated to the right of each interval position. High confidence (HC) genes are marked with red, while low confidence (LC) genes are colored with yellow. *HvMADS56 is* in green (Chr1-08). Full name of each gene is given in Table S4.

We identified the DNA and protein sequences of 108 different *HvMADS-box* genes located on all seven barley chromosomes; 13 on chromosome (chr) 1H, 14 on chr 2H, 17 on chr 3H, 9 on chr 4H, 13 on chr 5H, 19 on chr 6H and 23 on chr 7H (Figure 2). The gene annotation results showed that 30 of them are *Agamous-like MADS-box* transcription factors, 7 are K-box region and MADS-box transcription factor family proteins, and two are PISTILLATA-like MADS-box transcription factors. Fifty-one genes out of the 108 genes are orthologous to *MADS-box* genes in Arabidopsis or rice (or both). The other 57 genes could be unique to barley or yet unknown in other plants like Arabidopsis or rice (Table S3).

### 2.3. Phylogenetic Analysis of HvMADS-box Genes 

**Figure 3:**
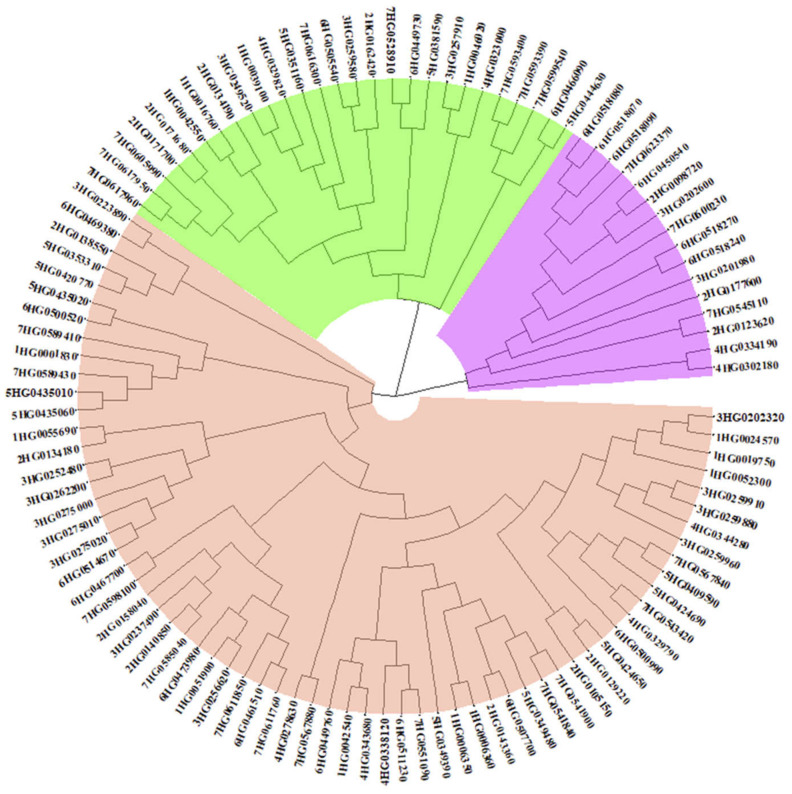
Phylogenetic tree obtained for the 108 *HvMADS-box* genes. A total of 108 protein sequences were identified and annotated as *MADS-box* transcription factors. The genes names in the tree derived from their names in the barley reference genome, e.g., 5HG0409590 corresponds to HORVU.MOREX.r2.5HG0409590.

We performed a phylogenetic analysis of the 108 *HvMADS-box* genes in barley based on their protein sequences. The results showed that these genes can be separated into three clades. The small clade includes 16 genes with 13 high confidence and 3 low confidence genes. The middle clade includes 27 genes with 16 high confidence and 11 low confidence genes. The remaining 65 genes make up the large clade, including 57 high confidence and 8 low confidence genes (Figure 3). The small clade (in purple color) includes *HvMADS-box* gene’ class *AGL80* and one gene belonging to *AGL65*. The middle clade is divided into five sub-clades and includes many of the unknown yet *MADS-box* genes either in barley, rice or Arabidopsis. The last clade includes all *HvMADS-box* genes, which have been assigned a function through studies in barley or through studies with their orthologous proteins in rice and Arabidopsis (Figure 3 and Table S3).

### 2.4. In Silico Analysis of Barley HvMADS-box Genes Expression

**Figure 4:**
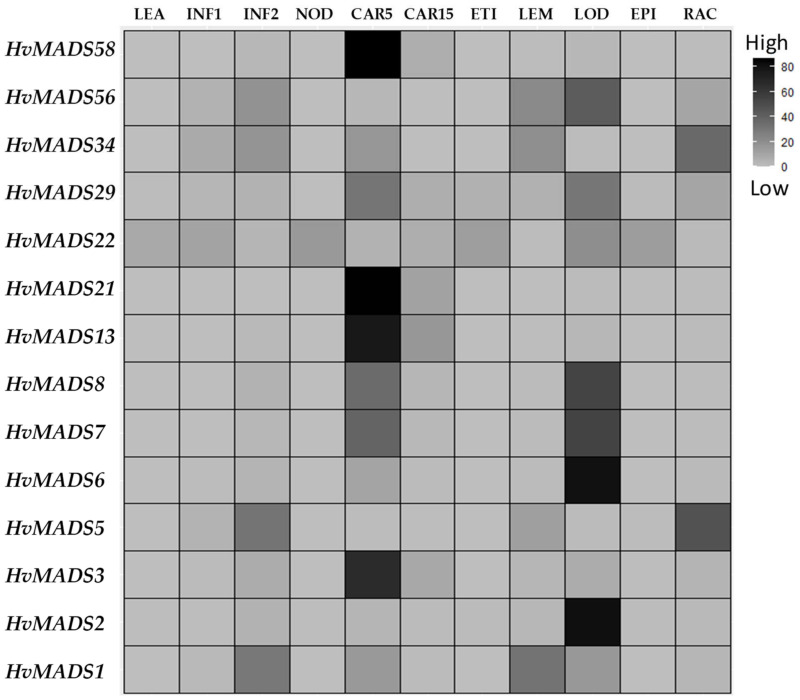
Clustering analysis of 14 *HvMADS-box* transcription factor gene transcripts in different barley tissues. LEA, leaves 17 days after planting (DAP); developing inflorescence 5mm (INF1, about 30 DAP); developing inflorescence 10–15mm (INF2, about 50 DAP); developing tillers (NOD), developing grain 5 days after anthesis (CAR5), developing grain 15 days after anthesis (CAR15), etiolated seedling (ETI), inflorescence lemma (LEM), inflorescence lodicule (LOD), epidermal strips (EPI), inflorescences rachis (RAC). Raw data from the https://apex.ipk-gatersleben.de/apex/f?p=284:10 website (accessed on 9 November 2021).

In order to understand how the different barley *HvMADS-box* transcription factors regulate floral initiation, development and growth, the Barlex database (https://apex.ipk-gatersleben.de/apex/f?p=284:10, accessed on 9 November 2021) was explored [25]. The Barlex database contains information on expression of genes in a wide range of barley tissues such as; 4-day embryos (EMB), roots from seedlings (ROO1), shoots from seedlings (LEA), young developing inflorescence (INF1), developing inflorescence (INF2), developing tillers (NOD), developing grains (CAR5), developing grain (CAR15), etiolated seedling (ETI), inflorescence lemma (LEM), inflorescence lodicule (LOD), dissected inflorescences palea (PAL), epidermal strips (EPI), inflorescences rachis (RAC), roots 28 days after planting (ROO2) and senescing leaves (SEN). It was found that 32 *HvMADS-box* genes were not expressed in any of the tested tissues, while 25 genes were expressed in all tissues. The remaining 51 genes showed different levels of expressions in one or more of the tested tissues (Table S3). Interestingly, none of the genes in the small clade showed any expression in the tested tissues except gene *AGL65,* which was expressed in all tissues. Of the genes in the second clade, eight showed no expression, six were expressed in all tissues, and the remaining 13 genes were expressed in one or more tissues. In the large clade, 12 genes showed no expression, 18 genes were expressed in all tissues, while the remaining 35 genes showed expression in one or more tissues (Table S3). In our study, we focused on the 14 *HvMADS-box* genes, which showed a high expression profile in the tissues or organs of the barley inflorescence (Figure 4). The results showed that *HvMADS1*, *3, 6, 7, 8, 13, 21, 29, 34* and *58* are specifically highly expressed in CAR5, while *MADS2, 6, 7, 8, 22, 29* and *56* are found in LOD and *MADS1, 5, 34* and *56* are highly presented in INF2.

### 2.5. MADS-box Genes Polymorphism and Lateral Spikelet Development

*MADS-box* genes, which were expressed mainly in tissues related to spikelet development, identified by our in silico analysis, were re-sequenced in all 30 lines in the present study to investigate the DNA sequence polymorphism that may affect spikelet development among these lines. The sequence variations in these genes showed polymorphism was largely absent among the tested lines except for *HvMADS3, 6, 8, 34, 21* and *56* (Table 2).

In *HvMADS3* (*HORVU.MOREX.r2.3HG0202320*), the results showed nucleotide substitutions from G to A in *def.4* at nucleotide position 84. This single nucleotide polymorphism (SNP) did not change the protein. In contrast, a G to C substitution at nucleotide position 104 in HOR1555 and HOR7041 was a nonsynonymous SNP causing a change from serine to threonine (Table 2). The sequence polymorphism observed in *HvMADS6* (*HORVU.MOREX.r2.6HG0500990*) was an 18 bp deletion from nucleotide 627 to 644 in HOR6211 and HOR6407 and a 21 bp deletion from nucleotide 627 to 647 in HOR10166. From the sequence data of *HvMADS8* (*HORVU.MOREX.r2.5HG0409590*), we found a GGCGGCGGCGGC insertion in *cv.* Bonus in the promotor region before the ATG start codon. In addition, a synonymous SNP from C to T in HOR7041 was observed.

The re-sequence data of *HvMADS34* (*HORVU.MOREX.r2.5HG0424690*) identified an SNP from G to A in the promotor region and a synonymous SNP from A to C at nucleotide position nine in HOR1555 and HOR7191. Re-sequencing of *HvMADS21* (*HORVU.MOREX.r2.1HG0052300*) identified three synonymous SNPs from G to C at nucleotide position 123 in *cv.* Kristina, *def.4*, *def.5* and *def.6*, *semi-def.8*, *int-h.43* and *int-l.81*, and from A to G at nucleotide position 411 in *cv.* Kristina, *def.4*, *semi-def.5*, *int-h.43*, *int-i.39* and HOR10166 and from G to T at nucleotide position 573 in HOR1555, HOR7191 and HOR7041. In addition, two insertions were observed: GGC at nucleotide positions 702-704 in HOR1555, HOR7191 and HOR7041 and C at nucleotide position 702, causing a frameshift and immature stop codon in *cv.* Foma, *semi-def.5, semi-def.6*, *int-c.5*, *int-e.20*, *int-f.19* and *hex-v.3*. The results of re-sequence *HvMADS56* (*HORVU.MOREX.r2.1HG0042540*) revealed a nonsynonymous SNP from T to A at nucleotide position 83, causing a change from leucine to glutamine in *semi-def.4, semi-def.5* and *semi-def.6*, a synonymous SNP from C to A at nucleotide position 105 in *def.4, def.5* and *def.6*, *semi-def.8*, *int-h.43*, *int-i.39*, HOR10166, HOR1555, HOR6211, HOR6407, HOR7191 and HOR7041. In addition, the results showed a G deletion at nucleotide position 321, causing a frameshift and immature stop codon in *cv.* Kristina, *def.5, def.6*, *semi-def.8*, *int-h.43*, *int-i.39*, HOR10166, HOR1555, HOR6211, HOR6407, HOR7191 and HOR7041, as well as a 67 bp deletion in *semi-def.1* (Table 2). The SNP analysis revealed a correlation between lines with contrasting lateral spikelet phenotype and the SNPs and in/del polymorphisms detected in the *HvMADS6* and *HvMADS56*. Therefore, the G deletion at nucleotide position 321 in *HvMADS56* may be responsible for the significant lateral spikelet reduction in *cv.* Kristina, *def.5, def.6, Semi-def.8*, *int-h.43*, *int-i.39*, while the 67 bp deletion may account for the effect seen in *semi-def.1*. The lateral spikelet reduction in the three lines, HOR10166, HOR6211 and HOR6407, might be due to the 18 bp deletion in HOR6211 and HOR6407 and the 21 bp deletion in HOR10166 in *HvMADS6* as well as the G deletion at nucleotide position 321 in all of the three lines in *HvMADS56.*

## 3. Discussion

The aim of the present study was to further elucidate the genetic basis of barley spike and spikelet development to increase seed yield. We used 30 different lines with variations in lateral spikelet formation. Genetic and phenotypic variation remains a cornerstone in plant breeding. Understanding the mechanisms of the relative fertility of central and lateral spikelets will provide novel solutions to enhanced grain yield in barley [11]. This could be achieved by increasing the number of seeds per spike which is a major goal of modern plant breeding programs [26]. The *MADS-box* genes family is considered to represent the first discovered transcription factor proteins [17,18]. In plants, MADS-box proteins are involved in various developmental processes during flowering [27,28,29] and are proposed to be the driving force behind floral diversity [19,20]. Therefore, a better understanding of their role and function in barley could help improve cereal breeding programs.

In the current study, we found a large variation among the tested lines for all central and lateral spikelet-related traits. All these traits had a broad-sense heritability of over 90% (Table 1 and Table S2), which confirmed that genetic effects are the major determinant of the phenotypic variance of these traits in barley. Our results are consistent with those obtained by [30,31], describing different fertility degrees for lateral spikelets in the intermedium-spike barley collection ranging from completely infertile (two-rowed like) to full fertile (six-rowed like). Our scanning electron micrographs of the tested lines revealed clear variation in the lateral spikelet development, especially at the awn primordia stage.

Kuijer et al. [23] analyzed 34 *MADS-box* genes and noticed that they regulate floret, spikelet and spike development in barley. Our phylogenetic analysis further complemented these results by revealing the highly conserved nature of 108 *HvMADS-box* genes in barley, more than half of which (76 genes) were expressed in different barley tissues. Expression data of many of these genes (Figure 4) from individual floral organs such as INF1, INF2, LEM, LOD and PAL suggested a role in floret and/or spikelet and spike development in barley. These results are consistent with those obtained by Schilling et al. [32] in wheat and Ciaffi et al. [33] in rice, maize and wheat. 

We re-sequenced 14 of the *HvMADS-box* genes to investigate their role in lateral spikelet development. Sequence analysis of the 30 barley lines identified *HvMADS6* and *HvMDS56* as likely regulators of lateral spikelet development (Table 1 and Table 2). The G deletion and the 67 bp deletion in *HvMADS56* and 18 bp and 21 bp deletions in *HvMADS6* were likely candidates for inducing the reduced stage of lateral spikelets in *cv.* Kristina, *def.5, def.6, semi-def.1, semi-def.8*, *int-h.43*, *int-i.39*, HOR10166, HOR6211 and HOR6407.

**Figure 5:**
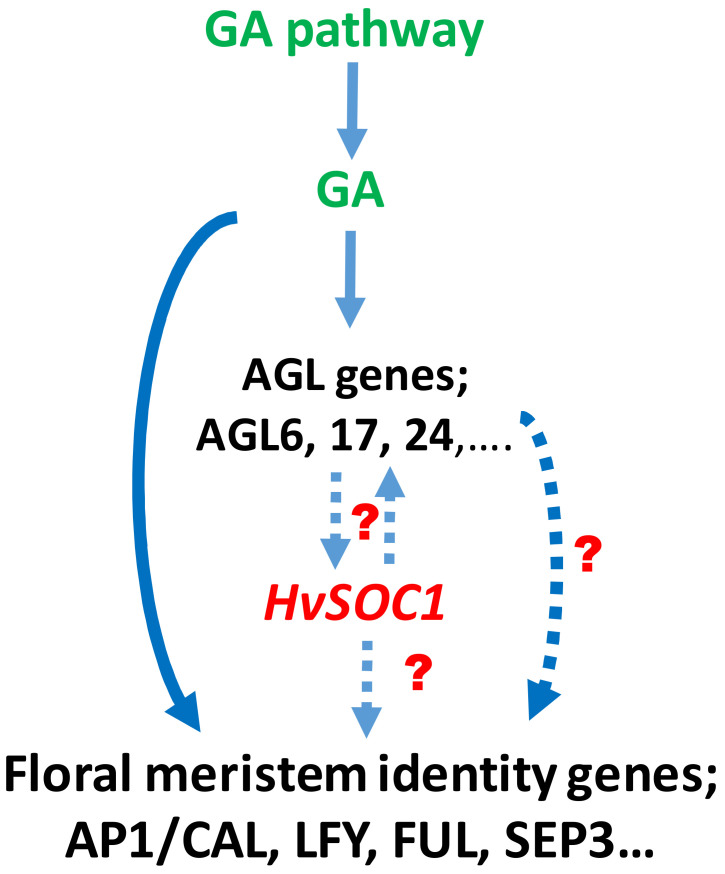
Suggested role of *HvMADS56* (*HvSOC1*) in regulating spikelet development in barley through its direct effect on the floral meristem identity genes or through stimulation of the AGAMOUS-like (AGL) genes activity. Hypothetical interactions that need further investigations in barley are indicated by dotted lines.

*HvMADS56,* which is located on barley chromosome 1H, is very similar to the putative ortholog in rice (*OsMADS56,* on chromosome 10) and putative ortholog in *Brachypodium* (Bradi3g32090, on chromosome 3) [34,35,36] composed seven exons and six introns [32]. The annotation result of these genes showed that they contain the characteristic K-domain and belong to the MADS-box (type II) family [37]. Blasting the DNA and protein sequences of *HvMADS56* to the NCBI database (https://blast.ncbi.nlm.nih.gov/Blast.cgi, accessed on 9 November 2021) revealed that, *HvMADS56* is similar to Arabidopsis *SUPPRESSOR OF OVEREXPRESSION OF CONSTANS (SOC1)* gene. Our in silico analysis showed that a maximum expression of *HvMADS56* (*HvSOC1*) was in inflorescence development organs INF2, LEM, LOD, PAL and RAC. These expression results were in agreement with what was found in Arabidopsis [38]. Papaefthimiou et al. [39] studied *HvSOC1-like1* and *HvSOC1-like2* and found that both are expressed at different stages during the reproductive phase portentous their possible insinuation in seed development. This clearly suggests an important role of *HvMADS56* (*HvSOC1*) in floret and spikelet development prior to the formation and setting of seeds. From the previous studies, SOC1 is known as a MADS-box transcription factor that is conserved and multifunctional protein in monocotyledons and dicotyledons [40,41,42,43,44,45], regulating flowering time, floral meristem patterning and determinacy [46,47,48]. It is also well known that hormones regulate floral organ patterning and spike and spikelet development in barley [10,16]. We suggest that the *HvMADS56* (*HvSOC1*) gene is functionally acting upstream of floral meristem identity genes in barley. In *Arabidopsis*, *Agamous-like* genes such as *AGL6*, *17* and *24* and *SOC1* interact and upregulate each other. The *SOC1* gene regulates *AP1*, *LFY*, *SEP3* and other flowering time genes (Figure 5). The effects of *SOC1* have also been reported in other species [49,50]. We hypothesize that the activation of the floral meristem identity genes and subsequent plant flowering in barley are affected by the gibberellic acid pathway either directly or indirectly through *HvSOC1* (Figure 5). This is in line with what is known about the role of *SOC1* and GA in regulating flowering and inflorescence development in *Arabidopsis* and Orchid [49,50].

## 4. Conclusions

In order to address the recent focus of crop breeding programs on flower development and its effect on yield, we studied the role of MADS-box proteins in inflorescence development in barley. Our results highlight the importance of *HvMADS56* in lateral spikelet development as well as floret and spikelet development prior to the formation and setting of seeds. The loss-of-function of *HvMADS56* due to the two deletions (a G and a 67 bp deletion) might cause the reduction and late spikelet development in some of the tested lines in this study. We also found that some other lines showed a reduction in lateral spikelet size, but they did not carry a polymorphism at the tested *MADS-box* genes. Thus, we conclude that *HvMADS56* (*HvSOC1*) not only plays an important role in floret and spikelet development but also other novel genes might be involved as well. Further investigations will be needed to solidify this conclusion and identify the relation between *HvMADS56* and the other genes underlying the spike and spikelet development in barley.

## 5. Materials and Methods

### 5.1. Plant Materials

Seeds of 30 spring barley lines (mutants, accessions and cultivars) with variation in lateral spikelet size were studied; two-rowed (4 lines; Foma, Bowman, Bonus, Kristina), *deficiens* (5 lines; *def*.*2*, *def.4* to *def.6 and def.8*), *semi-deficiens* (5 lines; *semi-def.1, semi-def.4, semi-def.5, semi-def.7* and *semi-def.8*), *intermedium* (*int*) mutants (9 lines; *int-a.1*, *int-c.5*, *int-e.4*, *int-e.20*, *int-f.19*, *int-h.43*, *int-i.39*, *int-l.81*, and *int-m.85*), *hexachiton* mutants (*hex-v.3*) and 6 accessions from the natural barley collection classified as *intermedium* barley [9], barley accessions (*Hordeum vulgare* L. convar. *intermedium* (Körn.) Mansf.). All the lines were obtained from NordGen (the Nordic Genetic Resource Center, Alnarp, Sweden), except the 6 lines of the natural collection that were obtained from the German Federal ex situ Genebank hosted at IPK Gatersleben, Germany (Table S5). 

### 5.2. Growth Conditions and Spike Phenotyping

Three replicates from each accession of the 30 lines were germinated and grown in the greenhouse facilities at the Department of Biology, Lund University, Lund, Sweden, between March and August 2019. The plants were grown in potting soil from SW Horto, Sweden (swhorto.se, article number 744704) in 1.5-L pots (a single plant/pot, 14 cm diameter and 14 cm height) under long-day conditions, 16 h light/8 h dark and temperature 20 ± 2 °C during the day and 16 ± 2 °C during the night. After harvest, the main culm spike of each plant was scored for central and lateral seed size; seeds weight, seed length, seed width, seed area, thousand seed weight, as well as plant seed number and plant seed weight, plant height (from the soil surface to the base of the spike), spike length (from the base to the tip of the spike without awns) and number per plant and number of tillers per plant. In addition to the lines which did not show fertility for the lateral spikelets, spikelet weight, spikelet length, spikelet width and spikelet area of 20 lateral florets from ten rachis nodes from the middle of the spike were measured after harvesting. The traits were measured with a MARVIN Seed Analyzer (GTA Sensorik, Neubrandenburg, Germany).

### 5.3. Scanning Electron Microscopy

Immature spike tissues (from five or more plants) at different spike developmental stages; triple mound, glum primordia, stamen primordia, lemma primordia and awn primordia were used for scanning electron microscopy (SEM) (Hisco Europe, Ratingen, Germany), which was conducted as previously described by Lolas et al. [51].

### 5.4. Genomic DNA Isolation, Amplification and Sequencing

Leaf samples were collected in 96-well plates from seedlings with 2–3 leaves. Genomic DNA extraction and polymerase chain reaction (PCR) amplifications were performed as described in Matyszczak et al. [52]. For each of the tested MADS-box genes in this study, primer pairs were designed to obtain sequence data for the whole gene by Sanger sequencing. Primer sequences, annealing temperatures and fragment lengths for all genes are provided in Table S6. PCR amplification profile with initial denaturation step: 3 min at 96 °C followed by the main program for 40 cycles at 96 °C for 40 s, 60 °C for 40 s and extension at 72 °C for 2 min, followed by a final extension for 12 min at 72 °C. The PCR products were tested on 1% agarose gel. For Sanger-sequencing, 2 µL of Exoprostar was added to 5 µL of the PCR products. The mixture was incubated at 37 °C for 15 min followed by 80 °C for 15 min. After that, 8 µL H_2_O and 2 µL of 10 μM primer were added to the Mixture and sent to the sequencing service offered by Eurofins Genomics, Germany.

### 5.5. Sequence Analysis and Sequence Homology Searches

Sequencher 5.2.3 DNA sequence assembly software (Gene Codes Corporation) was used for DNA sequencing analysis, quality score assignments and construction of contigs. Multiple sequence alignments were carried out using Clustal Omega (https://www.ebi.ac.uk/Tools/msa/clustalo/, accessed on 9 November 2021). Barley DNA sequences of the MADS-box genes with respective genetic and physical locations were extracted from [53,54] using the barley genome explore (Barlex) (https://apex.ipk-gatersleben.de/apex/f?p=284:10::::::, accessed on 9 November 2021). The positions of the MADS-box genes on all barley chromosomes were plotted using the R package ChromoMap [55].

### 5.6. Phylogenetic Analysis

MADS-box protein sequences were isolated using the barley genome explorer (Barlex) (https://apex.ipk-gatersleben.de/apex/f?p=284:10::::::, accessed on 9 November 2021). Protein sequences (108) were isolated and annotated as the *MADS-box* transcription factor. A multiple sequence alignment was performed using the R-package DECIPHER [56], and then a circular phylogenetic tree was generated using the R-package ggtree [57].

### 5.7. Expression Data Analysis

Transcript data of the *MADS-box* genes for the different barley plant tissues were isolated from the https://apex.ipk-gatersleben.de/apex/f?p=284:10:::::: website as FPKM—fragments per kilobase of exon model per million reads mapped (accessed on 9 November 2021). Source: [25].

### 5.8. Statistical Analysis

The analysis of variance (ANOVA) of a randomized complete block design (RCBD) experiment with three replications of the agronomic and central spikelet-related traits and with four replications of the lateral spikelet-related traits was performed using SAS software v.9.2 with PROC GLM procedure [58]. Means were compared by a Fisher’s least significant difference (LSD) procedure at 0.05 level of significance. Broad-sense heritability (H_b_) estimates were calculated under control according to [59].

## Figures and Tables

**Table 1:** The statistical analysis of the lateral spikelet traits in the set of 30 barley lines used in this study. Mean squares (MS) of lateral spikelet weight (LSW), lateral spikelet width (LSWi), lateral spikelet length (LSL), lateral spikelet area (LSA) and thousand seed or spikelet weight (TSW) in the tested lines.

			Mean Squares				
			LSW (g)	LSWi (mm)	LSL (mm)	LSA (mm^2^)	TSW (g)
			Means of Tested Lines				
***Two-rowed***	Bonus		0.0004	1.37	9.22	7.20	0.37
	Bowman		0.0005	1.69	8.49	6.61	0.54
	Foma		0.0005	1.62	9.37	7.63	0.48
	Kristina		0.0002	0.98	6.87	3.79	0.19
	**Average**		**0.0004**	**1.42**	**8.49**	**6.31**	**0.40**
***Deficiens***	*def.2*		0.0001	0.54	3.46	0.97	0.07
	*def.4*		0.0001	0.64	5.12	1.52	0.09
	*def.5*		0.0001	0.88	6.46	2.49	0.14
	*def.6*		0.0002	1.09	5.12	1.75	0.17
	*def.8*		0.0002	1.00	6.4	2.80	0.16
	**Average**		**0.0001**	**0.83**	**5.31**	**1.91**	**0.13**
***Semi-deficiens***	*semi-def.1*		0.0001	0.89	6.75	3.20	0.15
	*semi-def.4*		0.0002	0.97	5.86	3.00	0.17
	*semi-def.5*		0.0001	0.87	6.58	2.56	0.15
	*semi-def.7*		0.0002	0.91	7.38	3.17	0.17
	*semi-def.8*		0.0002	0.92	6.79	3.09	0.18
	**Average**		**0.0002**	**0.91**	**6.67**	**3.00**	**0.16**
***Intermedium* mutants**	*int-a.1*		0.0014	1.81	11.22	10.99	1.39
	*int-c.5*		0.0029	2.28	10.18	13.99	2.9
	*int-e.20*		0.0007	1.44	9.67	8.34	0.72
	*int-e.4*		0.0026	2.15	10.41	12.47	2.59
	*int-f.19*		0.0009	1.45	12.58	9.28	0.94
	*int-h.43*		0.0003	1.33	6.94	4.76	0.32
	*int-i.39*		0.0004	1.13	8.51	5.63	0.41
	*int-l.81*		0.002	2.23	10.71	12.81	2.02
	*int-m.85*		0.0007	1.81	10.18	8.42	0.7
	**Average**		**0.00132**	**1.74**	**10.04**	**9.63**	**1.33**
***Intermedium barley***	HOR1555		0.0416	4.12	9.77	26.84	41.56
	HOR6211		0.0008	1.52	9.15	8.01	0.8
	HOR7191		0.0477	4.23	10.11	27.93	47.74
	HOR10166		0.0026	1.73	12.04	13.83	2.57
	HOR6407		0.0007	1.19	10.13	6.79	0.75
	HOR7041		0.0227	3.43	14.55	32.13	22.71
	**Average**		**0.0194**	**2.70**	**10.96**	**19.26**	**19.36**
***Hexastichon mutant***							
	***hex-v.3***		0.0224	3.15	11.22	19.04	22.37
	LSD		4.2E-06	0.0841	0.6501	0.0324	0.0421
	Mean		0.0051 g	1.67 mm	8.78 mm	9.16 mm2	5.13 g
	Minimum		0.0001 g	0.54 mm	3.46 mm	0.97 mm2	0.07 g
	Maximum		0.0477 g	4.23 mm	14.55 mm	32.13 mm2	47.74 g

**Table 2:** Sequence variations and polymorphism at *Hv-MADS3, Hv-MADS6, HvMADS8, HvMADS34, HvMADS21 and HvMADS56,* including the nucleotide (nt) position of each among the tested lines.

	*HvMADS3*		*HvMADS6*	*HvMADS8*		*HvMADS34*		*HvMADS21*					*HvMADS56*			
	G/A (Glu/Glu) nt 84	G/C (Ser/Thr) nt 104	Del nt (627-647)	Ins in promotor	C/T (Asn/Asn) nt 285	G/A promotor	A/C (Arg/Arg) nt 9	G/C (Ala/Ala) nt 123	A/G nt 411 (Gly/Gly)	G/T nt 573 (Gly/Gly)	GGC Ins nt 702-704	C Ins at nt 702	T/A (Leu/Gln) in nt 83	A/C (Leu/Leu) in nt 105	G del at nt 321	67 bp del
**Bowman**	G	G			C	G	A	G	A	G			T	C		
**Bonus**	G	G		4x GGC	C	G	A	G	A	G			T	C		
**Foma**	G	G			C	G	A	G	A	G		C	T	C		
**Kristina**	G	G			C	G	A	C	G	G			T	C	-	
***def.2***	G	G			C	G	A	G	A	G			T	C		
***def.4***	A	G			C	G	A	C	G	G			T	A		
***def.5***	G	G			C	G	A	C	A	G			T	A	-	
***def.6***	G	G			C	G	A	C	A	G			T	A	-	
***def.8***	G	G			C	G	A	G	A	G			T	C		
***semi-def.1***	G	G			C	G	A	G	A	G			T	C		-67-
***semi-def.4***	G	G			C	G	A	G	A	G			A	C		
***semi-def.5***	G	G			C	G	A	G	G	G		C	A	C		
***semi-def.7***	G	G			C	G	A	G	A	G		C	A	C		
***semi-def.8***	G	G			C	G	A	C	A	G			T	A	-	
***int-a.1***	G	G			C	G	A	G	A	G			T	C		
***int-c.5***	G	G			C	G	A	G	A	G		C	T	C		
***int-e.4***	G	G			C	G	A	G	A	G			T	C		
***Int-e.20***	G	G			C	G	A	G	A	G		C	T	C		
***Int-f.19***	G	G			C	G	A	G	A	G		C	T	C		
***int-h.43***	G	G			C	G	A	C	G	G			T	A	-	
***int-i.39***	G	G			C	G	A	G	G	G			T	A	-	
***int-l.81***	G	G			C	G	A	C	A	G			T	C		
***int-m.85***	G	G			C	G	A	G	A	G			T	C		
***hex-v.3***	G	G			C	G	A	G	A	G		C	T	C		
**HOR10166**	G	G	-21-		C	G	A	G	G	G			T	A	-	
**HOR1555**	G	C			C	A	C	G	A	T	GGC		T	A		
**HOR6211**	G	G	-18-		C	G	A	G	A	G			T	A	-	
**HOR6407**	G	G	-18-		C	G	A	G	A	G			T	A	-	
**HOR7191**	G	G			C	A	C	G	A	T	GGC		T	A		
**HOR7041**	G	C			T	G	A	G	A	T	GGC		T	A		

## Data Availability

Data is contained within the article and Supplementary Materials.

## Supplementary Materials

The following are available online at https://www.mdpi.com/article/10.3390/plants10122825/s1. Table S1: Summary statistics of the agronomic, central spikelets and lateral spikelets related traits in the 30 tested lines; Table S2: The statistical analysis of variance, coefficient of determination (R^2^), broad-sense heritability (H_b_) and coefficient of variation (CV%) of the agronomic traits, central spikelet traits and lateral spikelet traits in the set of 30 barley lines used in this study; Table S3: List of MADS-box genes including gene ID, gene sequence, protein sequence, tissue of expression, chromosomal position and start and end of each gene on the barley chromosomes; Table S4: Genes ID for the map in Figure 2; Table S5: List of tested lines used in the study; Table S6: Primer sequences, annealing temperatures and fragment lengths for the tested genes.
